# Nurses’ implicit and explicit attitudes towards transgender people and the need for trans-affirming care

**DOI:** 10.1016/j.heliyon.2023.e20762

**Published:** 2023-11-02

**Authors:** Daniel W. Derbyshire, Tamsin Keay

**Affiliations:** a Research Fellow in Public Health Economics, European Centre for Environment and Human Health, University of Exeter, UK; b Assistant Professor in Nursing, Midwifery and Health Professions, Faculty of Health and Life Sciences, Coventry University, UK

**Keywords:** Transgender, Nursing education, Implicit bias, trans-affirmative education, Diversity, Nurse attitudes

## Abstract

**Background:**

The primary objective of this study is to investigate the implicit and explicit attitudes of healthcare professionals – in particular nurses – towards transgender people. This is especially important in light of recent calls for improved *trans*-affirming care provision by healthcare professionals to generate quality healthcare access and outcomes for transgender people.

**Methods:**

We use publicly available data from the transgender version of the Implicit Association Test from 2020 to 2022. We focus on differences between three subsets of participant: (1) non-healthcare professionals (N = 177,810), (2) non-nursing healthcare professionals (N = 22,443) and (3) nursing healthcare professionals (N = 11,996). We present the results of parametric statistical tests (t-tests) and an ordinary least squares regression, to analyse the robustness of our results when controlling for a host of sociodemographic characteristics.

**Results:**

We find that non-healthcare professionals have significantly lower implicit bias towards transgender people compared to healthcare professionals. Further, within healthcare professionals, we find nurses have significantly higher implicit bias towards transgender people compared to non-nurses. We show how implicit bias and explicit attitudes are highly correlated. Further, we provide evidence that healthcare professionals – but in particular nurses – conflate sex and gender identity.

**Conclusion:**

Whilst nurses continue to have higher levels of implicit and explicit bias towards transgender people there remains a need to globally establish additional enhanced *trans*-affirming care training provision for nursing and medical students.

## 1 Introduction

Transgender people are individuals whose gender identity differs from their sex assigned at birth. While the terms “sex” and “gender” are repeatedly coalesced, sex refers to one's sex, gender denotes personal and exclusive self-identifications and expression [1]. According to the 2021 UK Census, there are 262,000 transgender people aged 16 or over in England and Wales (0.5 % of the population), although 6 % of respondents chose not to answer this question and therefore this figure may be an under-representation [2]. In the US, a similar figure of 0.5 % of the population aged 18 or over (1.3 million people) identified as transgender, according to the Behaviour Risk Factor Surveillance Survey, although the figure is higher for young people aged 13 to 17 at 1.4 % (300,000 people) according to the Youth Risk Behaviour Survey [3].

According to the 2015 United States Transgender Survey, transgender individuals reported negative experiences in healthcare, such as verbal harassment or repudiation of treatment because of their gender identity. Worryingly, 24 % reported that they had to teach their medical providers about transgender access to care, and 23 % avoided compulsory medical intervention because they were concerned that they would be mistreated due to their gender identity [4]. This concern is not unwarranted since transgender people have reported being misgendered in healthcare settings [5]. Health-avoiding behaviours due to expectations of discrimination are a common experience among transgender individuals [6] and where transgender people can access care, many individuals are apprehensive that health care professionals (HCPs) will act in discriminatory ways, be unsupportive or provide unprofessional care [7]. Key topics within transgender health care include gender transitioning, mental health, sexual health and access to care [8].

In one qualitative study, undergraduate nursing students were interviewed about gender inclusive understanding. Just 5 % reported using gender inclusive constructs, 44 % were unaware of inclusive terms, 37 % did not understand what a gender inclusive concept was and there was a recurrent theme of confusing sex and gender identity [9]. Another study found that HCP knowledge of transgender health care was negatively associated with transphobic attitudes, suggesting the issue is attitudinal as well as educational [10]. Although student nurses expressed a strong interest in learning about transgender healthcare [11], HCPs reported lacking both the confidence and knowledge to provide adequate care to transgender patients [12]. This may be due to the lack of transgender content in both pre- and post-registration curricula. For example, nursing programs typically feature less than 3 h of teaching content related to LGBT healthcare [13], although there have been efforts to address this [14]. There is evidence that training can improve both attitudes towards transgender people and knowledge regarding transgender healthcare [15].

Time has been allocated to make a concerted effort to provide comprehensive, transgender specific health-care education, concurrently with associated global initiatives such as the Standards of Care for the Health of Transsexual, Transgender, and Gender Nonconforming People [16]. *Trans*-affirming care is characterised by patient-led care, a *trans*-affirming culture and *trans*-competent providers [17] and therefore has medical, social, psychological and behavioural facets [18,19]. This drive for *trans*-affirming care has included attempts to introduce innovations in education relating to transgender health care including improved educational models and content [20,21] and the use of simulation as a pedagogical tool [22,23]. Simulation scenarios could range from nursing students working with high-fidelity manikins to inter-professional education in a hospital setting and the use of additional service users from the transgender community.

In this study, we seek to investigate differences between HCPs and non-HCPs in implicit and explicit attitudes towards transgender people and explore how HCPs can address implicit bias to improve health care experiences for transgender individuals. We further distinguish between HCPs who are nurses and those who are not. We use publicly available data from Project Implicit (https://implicit.harvard.edu/implicit/) on the Transgender Implicit Association Test. We begin by outlining the Implicit Association Test procedure and our methods for analysing the data before describing the results and providing a discussion of the implications and limitations of our study.

## 2 Methods

### 2.1 Procedure

The Implicit Association Test (IAT) is a widely validated measure of implicit attitudes towards a range of personal characteristics including race, sexuality and gender [24]. Multiple meta-analyses have found the IAT to have predictive power beyond explicit attitudinal measures [[25], [26], [27]], although the validity of the IAT remains a contested subject in the literature [28,29]. Further, studies have shown that IAT results are difficult for participants to deliberately manipulate [[30], [31], [32]]. The transgender IAT is a recently developed variant of the IAT launched in April 2020 that measures implicit attitudes towards cisgender and transgender people [33].

**Fig. 1:**
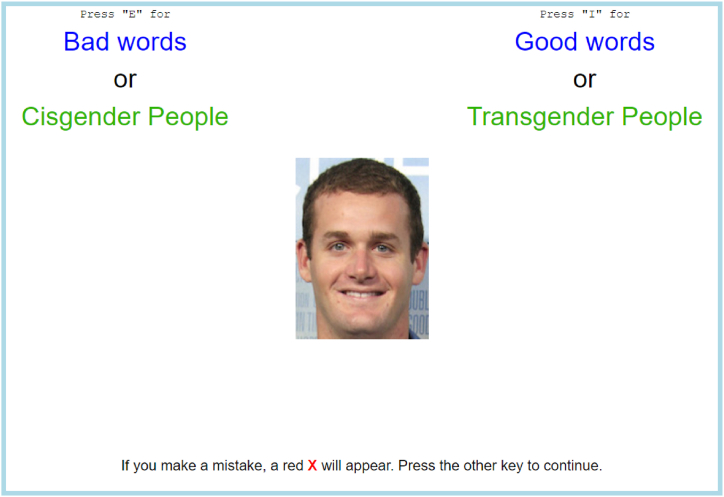
A screenshot of the transgender IAT procedure.

The IAT compares reaction times in assigning various stimuli (in the case of the transgender IAT, pictures of either cisgender or transgender celebrities) to pre-determined good/bad categories. The full set of stimuli used in the transgender IAT can be seen in Table 1. The order in which participants assign stimuli is randomised to control for order effects i.e. half of participants assign good-cisgender stimuli together first whilst the other half assign good-transgender stimuli together first. Participants assign one of the given stimuli 160 times across a series of blocks. A screenshot of the procedure can be seen in Fig. 1.

**Table 1:** Stimuli used for the transgender IAT.

Category	Items
Good	Nice, Pleasure, Laughter, Glorious
Bad	Nasty, Agony, Hurt, Rotten
Cisgender People	
Transgender People	

Note: All stimuli are taken from the Project Implicit demonstration site. The images are taken from Project Implicit's Open Science Framework repository at https://osf.io/na7d4/and are reproduced here in accordance with the CC0 license that the experimental materials are made available under.

The resulting measure from comparing the appropriate blocks of the IAT is referred to as a D-score which typically ranges from −2 (strongest possible pro-transgender implicit bias) to 2 (strongest possible anti-transgender implicit bias). Higher D-scores therefore relate to higher levels of anti-transgender bias [34]. Absolute values in excess of 0.15, 0.35 and 0.65 are referred to as slight, moderate and strong levels of implicit bias, respectively. Values between −0.15 and 0.15 are referred to as little to no implicit bias.

The procedure also involves a pre- and post-questionnaire that includes questions on sociodemographic characteristics and explicit attitudes towards transgender people including levels of agreement with various transphobic statements. This also includes the participant's occupation which we use to sort participants into HCP and non-HCP. We further distinguish between two subcategories of HCP in the analysis that follows; HCP-Nurses (i.e. Healthcare – Nursing and Home Health Assistants) and HCP-Non-Nurses (i.e. all other Healthcare occupation categories). Participants are also asked their explicit attitudes towards transgender people by choosing which of a series of statements they most agree with, ranging from *“I strongly/moderately/slightly prefer transgender people to cisgender people”* to *“I prefer transgender and cisgender people equally”* to *“I slightly/moderately/strongly prefer cisgender people to transgender people”*. Similarly, participants are asked to state how strongly they agree with a series of transphobic statements such as *“I believe a person can never change their gender”* that give additional insights into explicit attitudes.

### 2.2 Participants

The participants were people who visited the Project Implicit website (https://implicit.harvard.edu/implicit/takeatest.html) and completed the Transgender IAT in the years 2020, 2021 and 2022. Participants must be at least 18 years old and can be located anywhere in the world. Participants consented to their data being part of a publicly available dataset, which Project Implicit release on a yearly basis. In response to a question about what brought them to the Project Implicit website to undertake an IAT, around two thirds (66.75 %) of participants indicated that they were doing an assignment for work or school. Ethical approval for this secondary data analysis study was obtained from the University of Exeter Research Ethics Committee (ID 2420484).

### 2.3 Data

We used publicly available data from the transgender IAT for the years 2020 and 2022 that was not collected by the authors, but by Project Implicit [35]. We downloaded data and codebooks from the Project Implicit Open Science Framework repository (https://osf.io/fb29q/). The data is made available in the public domain under a CC0 License as per the Open Science Framework repository. We removed participants who did not complete the IAT procedure or answer all of the sociodemographic variables that we use as controls. We then removed participants who did not answer the question about their occupation. This left us with a total 212,249 participants in our sample. A total of 11,996 reported their occupation as being a nurse and a further 22,443 reported being some other kind of healthcare professional (HCP), leaving 177,810 non-HCP participants. We present the results of Cohen's *d* effect sizes and parametric t-tests to test for significant differences in terms of implicit and explicit attitudes towards transgender people [36]. We also present a regression analysis to control for other important demographic variation within our sample. Test statistics presented are t-statistics and associated p-values unless otherwise stated. All results are unchanged when performing non-parametric Mann Whitney U (rank sum) tests or alternative regression techniques. The coding for the analysis presented can be found at https://osf.io/hscya.

## 3 Results

### 3.1 Descriptive statistics

We begin by presenting some summary statistics related to our 212,249 observations. As can be seen in Table 2, 66 % of participants identified as female and 4 % identified as a non-binary gender. Further, 62 % had a university degree or higher in terms of education. The average age was 34 years and 77 % of participants were white. Table 2 also provides a breakdown between participants who indicated that their occupation was some form of healthcare professional and all other occupations. As can be seen, the subset of participants who are HCPs are significantly more educated and more likely to be female. They are also older and less likely to be white, though these effect sizes are relatively small compared to gender and education differences. Notably, only 4 % of HCPs reported being a non-binary gender, compared to 7 % for non-HCPs. While these differences are to a greater or lesser extent not surprising, there are significant differences across these two subsamples that should be taken as a caveat throughout the remainder of the results section. The regression analysis presented below controls for these subsample differences.

**Table 2:** (Sub-)Sample descriptive statistics.

Variable	Full Sample	Non-HCP	HCP-Non-Nurses	HCP-Nurses
White	0.77	0.77	0.76	0.79
Female	0.66	0.64	0.74	0.89
Non-Binary	0.04	0.04	0.03	0.01
Degree	0.62	0.61	0.75	0.57
Age	34.40	34.23	35.42	34.93

We construct three occupational categories; (1) non-HCP (N = 177,810) for participants indicating a non-healthcare based occupation, (2) HCP-Non-Nurses (N = 22,443) for participants indicating any healthcare occupation *except* ‘Nursing and Home Health Assistants’, and (3) HCP-Nurses (N = 11,996) for participants who specified their occupation as ‘Healthcare – Nursing and Home Health Assistants’.

### 3.2 Implicit and explicit bias towards transgender people

**Fig. 2:**
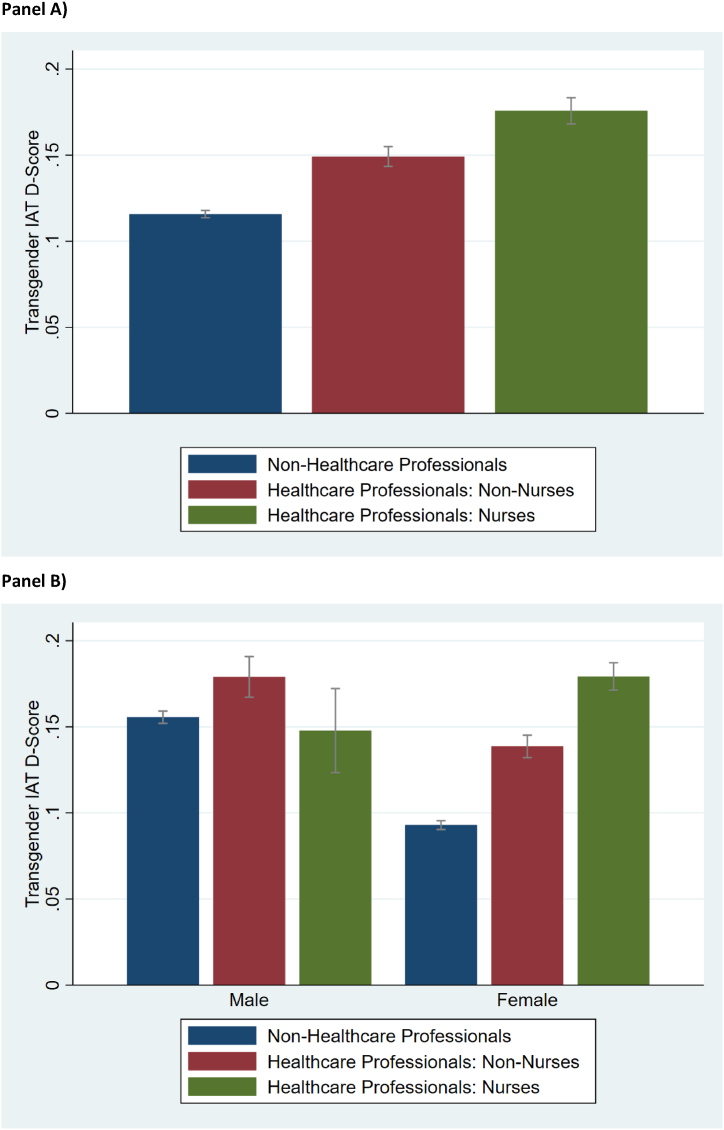
Transgender IAT D-scores by healthcare professional status for the full sample (panel A) and split by gender (panel B).

We begin by investigating differences in implicit bias between non-HCP, HCP-Non-Nurses and HCP-Nurse participants. Fig. 2 shows the average transgender IAT D-scores for each group, including the 95 % confidence intervals. HCP-Non-Nurses (mean 0.149, standard deviation (SD) 0.003) have significantly higher implicit bias towards transgender people compared to non-HCP (mean 0.116, SD 0.001; p < 0.001, *d* = −0.075). HCP-Nurses (mean 0.176, SD 0.004) also have significantly higher implicit bias than HCP-Non-Nurses (p < 0.001, *d* = −0.061) and non-HCP participants (p < 0.001, *d* = −0.135). In all cases the effect sizes are relatively small but significant. Further, whilst on average non-HCP have little to no implicit bias towards transgender people, HCP-Nurses have slight implicit bias (i.e. greater than 0.15) towards transgender people. We note that these results are robust to excluding college/university aged participants; we present a similar analysis for participants aged 23 or over in Appendix A1. Similarly, these results are robust to only focusing on female participants (since HCP are predominantly women). Fig. 2 also includes a breakdown between males and females. Male non-HCP have significantly higher implicit bias compared to female non-HCP (p < 0.001, *d* = 0.141). Similarly, male HCP-non-nurses have significantly higher implicit bias than female HCP-non-nurses (p < 0.001, *d* = 0.092). Conversely, male HCP-nurses have significantly lower implicit bias than female HCP-nurses (p < 0.001, *d* = −0.074). Again, all the effect sizes are relatively small.

**Fig. 3:**
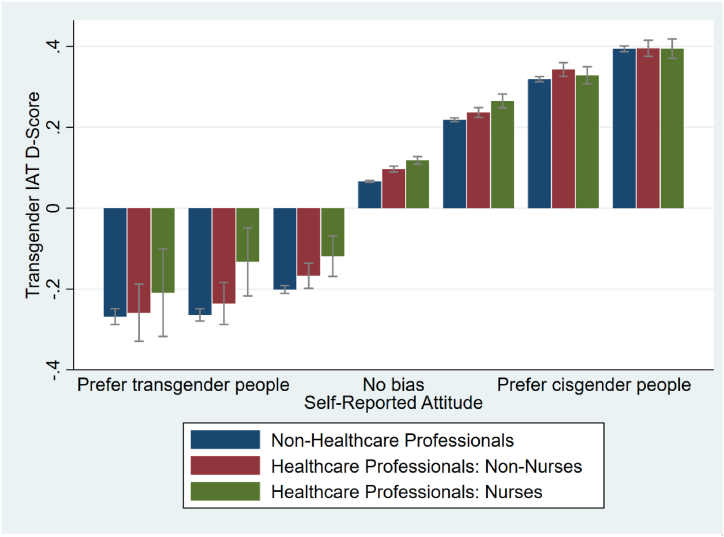
Transgender IAT D-scores and explicit attitudes towards transgender people by healthcare professional status.

The transgender IAT D-scores for every possible level of explicit attitudes (from strongly prefer transgender people to strongly prefer cisgender people) is shown in Fig. 3, again broken down between non-HCP, HCP-Non-Nurses and HCP-Nurses. As can be seen, there is a strong association between implicit and explicit attitudes towards transgender people, for all occupational categories including nurses. This 7-point scale gives an overall measure of the participants’ explicit attitudes towards transgender people. In terms of explicit attitudes, HCP-Nurses (mean 4.54, SD 0.009) have significantly worse explicit attitudes towards transgender people compared to both HCP-Non-Nurses (mean 4.48, SD 0.007; p < 0.001, *d* = −0.061) and Non-HCP (mean 4.44, SD 0.003; p < 0.001, *d* = −0.097). Therefore, the effect sizes for explicit biases are substantially lower than the effect sizes seen for implicit bias and the differences in explicit bias are relatively small in comparison.

## 4 Conflating sex and gender identity

Participants are asked a series of statements relating to transphobic viewpoints and asked to state their level of agreement with each statement along a 7-point scale from 1 = strongly disagree to 7 = strongly agree. As such, higher average scores relate to stronger levels of agreement with transphobic viewpoints. Table 3 gives a summary of the responses to a selection of these statements split according to whether a participant is an HCP. As can be seen, there are negligible differences in between HCP-non-nurses and non-HCP. However, HCP-Nurses are significantly more likely to agree with transphobic statements that conflate sex and gender identity such as *“I believe the male/female dichotomy is natural”* and *“I believe a person can never change their gender”* when compared to both non-HCP and HCP-Non-Nurses, although the effect sizes are small.

**Table 3:** Agreement with transphobic viewpoints for non-healthcare professionals (Non-HCP), non-nursing healthcare professionals (HCP-Non-Nurses) and nursing healthcare professionals (HCP-Nurses).

“I believe the male/female dichotomy is natural”					
	Average	N	95 % C.I.	Cohen's *d* (p-value)	
**Non-HCP**	3.71	58,908	[3.69, 3.73]	−0.026 (0.018)	
**HCP-Non-nurses**	3.76	7407	[3.72, 3.81]		−0.119 (<0.001)
**HCP-Nurses**	3.99	3964	[3.93, 4.05]		
**“I believe a person can never change their gender”**					
	**Average**	**N**	**95 % C.I.**	**Cohen's *d* (p-value)**	
**Non-HCP**	2.32	59,478	[2.30, 2.33]	<-0.001 (0.491)	
**HCP-Non-nurses**	2.32	7443	[2.27, 2.36]		−0.195 (<0.001)
**HCP-Nurses**	2.69	4016	[2.63, 2.76]		
**“I think there is something wrong with a person who says they are neither a man nor a woman”**					
	**Average**	**N**	**95 % C.I.**	**Cohen's *d* (p-value)**	
**Non-HCP**	2.56	59,448	[2.55, 2.58]	−0.020 (0.048)	
**HCP-Non-nurses**	2.60	7440	[2.56, 2.65]		−0.213 (<0.001)
**HCP-Nurses**	3.01	4006	[2.95, 3.07]		
**“A person's genitalia defines what gender they are, e.g., a penis defines a person as a man, a vagina defines a person as a woman”**					
	**Average**	**N**	**95 % C.I.**	**Cohen's *d* (p-value)**	
**Non-HCP**	2.97	59,473	[2.95, 2.99]	−0.006 (0.322)	
**HCP-Non-nurses**	2.96	7446	[2.91, 3.01]		−0.204 (<0.001)
**HCP-Nurses**	3.41	4016	[3.39, 3.48]		

Further, whilst both HCP-Nurses and HCP-Non-Nurses are more likely to say they've met a transgender person compared to non-HCP (p=<0.027), they are less likely to say they have either family (p=<0.085) or friends (p < 0.001) who are transgender when compared to non-HCP. This suggests that HCPs (both nurses and non-nurses) experience of interacting with transgender people may be largely confined to a work context.

### 4.1 Regression analysis

We now present the results of a regression analysis in order to investigate the robustness of the presented results to other important factors, in particular the sociodemographic differences between HCP (Nurses and Non-Nurses) and non-HCP highlighted above. A table presenting an overview of the variables used in the regression can be found in Appendix A2.

Table 4 presents the results of an ordinary least squares (OLS) regression featuring the participant's transgender IAT D-score as the dependent variable and utilising the variables described in TAppendix A2 as explanatory variables. As can be seen, all coefficients are significant at the 1 % level (except for having a degree) and have the expected sign. Factors associated with lower implicit bias towards transgender people are being white, being female, being non-binary and being more politically liberal. Conversely, factors associated with higher levels of implicit bias towards transgender people are explicit attitudes, age and higher levels of religiousness.

**Table 4:** Ordinary Least Squares (OLS) regression analysis using the transgender IAT D-score as the dependent variables with robust standard errors.

Dependent Variable: IAT D-score	Coefficient (Standard Error)	t-statistic (p-value)
HCP-Non-Nurses	0.022*** (0.002)	7.75 (<0.001)
HCP-Nurses	0.034*** (0.003)	9.00 (<0.001)
Explicit Attitude	0.083*** (<0.001)	87.85 (<0.001)
Age	0.005*** (<0.001)	69.74 (<0.001)
White	−0.036*** (0.002)	−16.44 (<0.001)
Degree	0.002 (0.002)	1.02 (0.309)
Female	−0.072*** (0.002)	−35.39 (<0.001)
Non-Binary	−0.229*** (0.005)	−46.16 (<0.001)
Religiousness	0.013*** (0.001)	12.50 (<0.001)
Political Identity	−0.040*** (0.001)	−62.94 (<0.001)
Constant	−0.169*** (0.007)	−23.58 (<0.001)
Sample Size	212,249	
R^2	0.158	

In terms of HCPs, we find a significant and robust effect that both HCP-Non-Nurses and HCP-Nurses have higher levels of implicit bias towards transgender people compared to non-HCP participants. Furthermore, the effect of being an HCP-Nurse is significantly higher than being an HCP-Non-Nurse in terms of implicit bias (p = 0.009). As such, our results show a significant and robust effect of HCPs – especially nurses – being associated with higher levels of implicit bias towards transgender people. Again, these results are robust to excluding college/university aged participants; we present the results of a regression with only participants aged 23 or over in Appendix A3.

## 5 Discussion

We used publicly available data for the Transgender IAT from 2020 to 2022 to investigate implicit and explicit attitudes towards transgender people and how they differ between health care professionals (HCPs) and non-HCPs. We further distinguished between nursing HCPs and other HCPs. We found that HCP-nurses have significantly more negative implicit attitudes towards transgender people compared to HCP-non-nurses and non-HCPs. Further, we found that HCP-nurses were more likely to agree with transphobic statements that conflate sex and gender identity when compared to both HCP-non-nurses and non-HCPs.

Our finding that HCP-nurses have higher levels of implicit bias towards transgender people may be related to a tendency to conflate sex and gender identity, as shown by higher levels of agreement with transphobic statements that conflate these two distinct concepts. This is despite relatively small differences in overall explicit attitudes as measured by the single-item explicit measure, suggesting that HCP may attempt to conceal or are unaware of their implicit attitudes. Previous research suggests that these implicit and explicit attitudes towards transgender people are predictors of policy views on issues such as bathrooms [37]. Nonetheless, research suggests this is not ‘simply’ a case of better education and that addressing attitudinal biases may be a prerequisite to successfully implementing educational interventions that result in improved understanding and knowledge of transgender health care issues [10]. Further, it has been acknowledged that nursing as a discipline has lagged behind other healthcare disciplines in adequately addressing issues related to culturally competent transgender health care [13,38] which is reflected in the results presented here. Cultural competency has been highlighted as a key component of effective *trans*-affirming care [39].

Nurses have an ethical and professional responsibility to treat all patients with dignity and respect. Therefore, it is crucially important for HCPs to gain awareness and understanding of transgender patients, and to provide *trans*-affirmative and sensitive care. To explore how implicit bias contributes to healthcare gaps experienced by transgender people globally, nurses must actively engage in self-reflection surrounding the position of power and privilege they control in the transgender patient/nurse relationship. Once nurses recognise their own biases and augment their knowledge about transgender individuals, they can proceed to provide *trans*-affirming healthcare as opposed to the traditional social and medical norms of healthcare [40]. Nurses who are naive about terminology related to gender may unconsciously exclude patients and their families. Gender inclusive forms allow people to be seen, heard and feel included in all aspects of healthcare. Experiences of such *trans*-affirming responses have been related to high levels of satisfaction among young people and their families attending clinics [41,42].

## 6 Limitations

The sample used for this study was a self-selected sample of people who chose to visit the Project Implicit website and chose to both begin and complete the Transgender IAT. As such, the sample may be subject to sample selection bias in terms of the demographics and IAT results of participants. However, it may be anticipated that people with particularly negative attitudes towards transgender people would avoid taking the Transgender IAT and the results presented here may therefore under-represent the extent of implicit bias towards transgender people. Further, and as noted above, the IAT in general is a measure that has been the subject of much discussion within the literature [28,29], with questions about the validity of the IAT measure as a predictor of explicit attitudes and/or behaviour. The generalisability of IAT results to real world attitudes and behaviours may therefore be limited.

## 7 Conclusion

Negative implicit and explicit attitudes towards transgender people are a barrier to the successful implementation of adequate *trans*-affirming care within health care settings, particularly in relation to nurses. Appropriate education for health care professionals – including nurses – around transgender health care issues should include attempts to address the conflation of sex and gender and persistent attitudinal barriers.

## Data Availability

The data used in this study can be obtained from the Project Implicit repository on Open Science Framework available at https://osf.io/fb29q/. The codes used for analysis are available from the author's OSF repository at https://osf.io/hscya.

## Ethical approval

Ethical approval for this research was granted by the 10.13039/501100000737University of Exeter Research Ethics Committee under application 2420484.

## CRediT authorship contribution statement

**Daniel W. Derbyshire:** Conceptualization, Formal analysis, Methodology, Software, Visualization, Writing – original draft, Writing – review & editing. **Tamsin Keay:** Conceptualization, Writing – original draft, Writing – review & editing.

## Declaration of competing interest

The authors declare that they have no known competing financial interests or personal relationships that could have appeared to influence the work reported in this paper.
