# Supplementary material for: Nurses’ implicit and explicit attitudes towards transgender people and the need for trans-affirming care
Source: Heliyon. 2023 Nov 2;9(11):e20762. doi: 10.1016/j.heliyon.2023.e20762 (PMC10722320; doi:10.1016/j.heliyon.2023.e20762)
Supplement: Multimedia component 1 [file mmc1.docx]

| **Table A1a: Overall effect sizes (Cohen’s *d*) for Transgender IAT D-scores** | | |
| --- | --- | --- |
|  | **HCP-Non-Nurses** | **HCP-Nurses** |
| **Non-HCP** | -0.075 | -0.134 |
| **HCP-Non-Nurses** | X | -0.061 |

| **Table A1b:** **Overall effect sizes (Cohen’s *d*) for Transgender IAT D-scores; participants aged 23 or over only** | | |
| --- | --- | --- |
|  | **HCP-Non-Nurses** | **HCP-Nurses** |
| **Non-HCP** | -0.096 | -0.178 |
| **HCP-Non-Nurses** | X | -0.105 |

| **Table A1c: Overall effect sizes (Cohen’s *d*) for Transgender IAT D-scores; female participants only** | | |
| --- | --- | --- |
|  | **HCP-Non-Nurses** | **HCP-Nurses** |
| **Non-HCP** | -0.052 | -0.143 |
| **HCP-Non-Nurses** | X | -0.070 |
