# Supplementary material for: Nurses’ implicit and explicit attitudes towards transgender people and the need for trans-affirming care
Source: Heliyon. 2023 Nov 2;9(11):e20762. doi: 10.1016/j.heliyon.2023.e20762 (PMC10722320; doi:10.1016/j.heliyon.2023.e20762)
Supplement: Multimedia component 2 [file mmc2.docx]

| **Table A2: Overview of variables used in regression analysis (N=212,249)** | | | |
| --- | --- | --- | --- |
| **Variable** | **Explanation** | **Mean** | **S.D.** |
| HCP-Non-Nurses | Dummy = 1 if HCP-Non-Nurse | 0.11 | - |
| HCP-Nurses | Dummy = 1 if HCP-Nurse | 0.06 | - |
| Explicit Attitude | Explicit attitude on a 7 point scale (higher values = higher anti-transgender bias). | 4.45 | 1.08 |
| Age | Participant’s age | 34.40 | 13.13 |
| White | Dummy = 1 if white | 0.77 | 0.42 |
| Degree | Dummy = 1 if has a degree | 0.62 | 0.49 |
| Female | Dummy = 1 if female | 0.66 | 0.47 |
| Non-Binary | Dummy = 1 if non-binary | 0.04 | 0.20 |
| Religiousness | Participant’s religiousness on a 4 point scale (higher values = more religious) | 1.91 | 0.97 |
| Political Identity | Participant’s political identity on a 7 point scale (higher values = more liberal) | 5.09 | 1.66 |
