# Supplementary material for: Nurses’ implicit and explicit attitudes towards transgender people and the need for trans-affirming care
Source: Heliyon. 2023 Nov 2;9(11):e20762. doi: 10.1016/j.heliyon.2023.e20762 (PMC10722320; doi:10.1016/j.heliyon.2023.e20762)
Supplement: Multimedia component 3 [file mmc3.docx]

| **Table A3: Ordinary Least Squares (OLS) regression analysis using the transgender IAT D-score as the dependent variables with robust standard errors; participants aged 23 or over only.** | | |
| --- | --- | --- |
| **Dependent Variable: IAT D-score** | **Coefficient (Standard Error)** | **t-statistic (p-value)** |
| HCP-Non-Nurses | 0.024*** (0.003) | 7.72 (<0.001) |
| HCP-Nurses | 0.037*** (0.004) | 8.91 (<0.001) |
| Explicit Attitude | 0.083*** (<0.001) | 77.12 (<0.001) |
| Age | 0.005*** (<0.001) | 60.26 (<0.001) |
| White | -0.037*** (0.002) | -15.02 (<0.001) |
| Degree | 0.001 (0.002) | 0.59 (0.556) |
| Female | -0.068*** (0.002) | -29.76 (<0.001) |
| Non-Binary | -0.250*** (0.006) | -40.50 (<0.001) |
| Religiousness | 0.012*** (0.001) | 10.06 (<0.001) |
| Political Identity | -0.041*** (0.001) | -57.99 (<0.001) |
| Constant | -0.162*** (0.008) | -19.94 (<0.001) |
| Sample Size | 167,368 | |
| R^2 | 0.150 | |
